# *MDR1* overexpression combined with *ERG11* mutations induce high-level fluconazole resistance in *Candida tropicalis* clinical isolates

**DOI:** 10.1186/s12879-018-3082-0

**Published:** 2018-04-10

**Authors:** Longyang Jin, Zhuorui Cao, Qi Wang, Yichen Wang, Xiaojuan Wang, Hongbin Chen, Hui Wang

**Affiliations:** 1 Department of Clinical Laboratory, Peking University People’s Hospital, No. 11 Xizhimen South Street, Xicheng District, Beijing, 100044 People’s Republic of China; 2 International Curriculum Center, The High School Affiliated to the Renmin University of China, Beijing, 100080 China

**Keywords:** *Candida tropicalis*, Fluconazole resistance, *MDR1*

## Abstract

**Background:**

Marked increases in fluconazole resistance in *Candida tropicalis* have been recently reported. In this study, the molecular mechanisms behind fluconazole resistance were investigated.

**Methods:**

Twenty-two *C. tropicalis* clinical isolates, including 12 fluconazole-resistant isolates and 10 fluconazole-susceptible isolates, were collected from a tertiary care teaching hospital in Beijing between 2013 and 2017. Antifungal susceptibility testing, multilocus sequence typing, *ERG11* amplification and sequencing, quantitative real-time reverse transcription-polymerase chain reaction (*ERG11*, *UPC2*, *MDR1*, and *CDR1*), and clinical data collection were performed for all *C. tropicalis* isolates.

**Results:**

Multilocus sequence typing revealed that the 10 fluconazole-susceptible isolates and 12 fluconazole-resistant isolates were divided into nine and seven diploid sequence types, respectively. Of the 12 patients with fluconazole-resistant isolates, six had been previously exposed to azole and four had a fatal outcome. Y132F and S154F amino acid substitutions in Erg11p were found in all fluconazole-resistant isolates except one. *MDR1* gene overexpression was identified in fluconazole-resistant isolates. In particular, seven high-level fluconazole resistant isolates (minimum inhibitory concentration ≥ 128 mg/L) and three pan-azole resistant isolates were identified. *CDR1*, *ERG11*, and *UPC2* gene expression levels in fluconazole-resistant isolates were not significantly different from the control isolates (*P* = 0.262, *P* = 0.598, *P* = 0.114, respectively).

**Conclusions:**

This study provides evidence that the combination of *MDR1* gene overexpression and *ERG11* missense mutations is responsible for high-level fluconazole resistance and pan-azole resistance in *C. tropicalis* clinical isolates. To the best of our knowledge, this is the first study investigating the relationship between *MDR1* gene overexpression and increased fluconazole resistance.

## Background

In recent years, *Candida* species have emerged as important causes of nosocomial infections leading to high morbidity and mortality, particularly in immunocompromised patients [1–3]. In China, the prevalence of *Candida tropicalis* is similar to that of *Candida parapsilosis*, making it the second most common non-*albicans Candida* species [4].

Currently, azole antifungal agents are widely used in the battle against clinical *Candida* infections [5]. Due to the lower risk of side effects and low cost associated with fluconazole, it has become one of the most commonly used antifungals [6]. Until recently, *C. tropicalis* was susceptible to azole and other antifungal agents. However, azole resistance in *C. tropicalis* has been recently observed, possibly owing to the extensive use of antifungals in medical institutions [7]. Pan-azole-resistant *C. tropicalis* isolates, which pose serious clinical challenges, have also been found in multiple countries [8–10]. Indeed, the widespread use of azoles has been deemed to be an independent risk factor for fluconazole resistance in *Candida* species [11]. In addition, *C. tropicalis* appears to develop fluconazole resistance much more rapidly than other *Candida* species under in vitro selection [12].

Azole resistance in *Candida* species has been associated with mutations in or overexpression of the *ERG11* gene or with upregulation of the *CDR1* and *MDR1* genes, the products of which are active transport pumps [13]. *ERG11* gene mutations and overexpression have been demonstrated to be responsible for azole resistance in *C. tropicalis* clinical isolates in China [14]. However, there is still insufficient evidence of the role of the *MDR1* and *CDR1* genes in azole resistance of *C. tropicalis* clinical isolates. Therefore, in order to better understand the molecular mechanisms underlying azole resistance in *C. tropicalis*, we obtained a collection of fluconazole-resistant *C. tropicalis* isolates from a tertiary care teaching hospital and evaluated them for efflux pump gene expression level as well as *ERG11* missense mutations. Our findings suggest that *ERG11* missense mutations and increased *MDR1* gene expression are highly correlated with fluconazole resistance in *C. tropicalis* clinical isolates.

## Methods

### Strains and clinical data collection

A total of 12 non-duplicated fluconazole-resistant (minimum inhibitory concentration [MIC], ≥8 mg/L) *C. tropicalis* isolates were retrospectively collected from hospitalized patients at Peking University People’s Hospital, a tertiary care teaching hospital with 1448 beds located in Beijing, China between 2013 and 2017. In the Peking University People’s Hospital, a high rate of resistance to fluconazole, with 20.30% resistant *C. tropicalis* isolates, was observed between 2013 and 2017. These isolates were recovered from various clinical specimens (excluding sputum samples) and presumptively identified by CHROMagar *Candida* (CHROMagar, Paris, France) and then confirmed by matrix-assisted laser desorption/ionization (MALDI-TOF) mass spectrometry (MS) (Bruker Daltonik GmbH, Bremen, Germany). Fluconazole-susceptible isolates (MIC, ≤2 mg/L) were randomly obtained from different wards between 2013 and 2017. All strains were stored at − 80 °C in 20% glycerol until use and maintained by biweekly passage on Sabouraud agar (Oxoid, Basingstoke, United Kingdom).

Clinical data and patient information were obtained retrospectively from medical records. Previous use of azole antifungal agents was defined as administration within 3 months prior to the isolation of *C. tropicalis*. Inpatient outcomes were assessed 30 days after *C. tropicalis* isolation*.* This study was approved by the Research Ethics Board at Peking University People’s Hospital.

### Antifungal susceptibility testing

Antifungal susceptibility testing was performed using Sensititre YeastOne™ YO10 methodology (Thermo Scientific, Cleveland, OH, USA) in accordance with the manufacturer’s instructions. MICs were determined after 24 h of incubation at 35 °C and interpreted according to species-specific Clinical & Laboratory Standards (CLSI) breakpoints [15]. *Candida krusei* ATCC 6258 and *C. parapsilosis* ATCC 22019 were used as quality controls.

### PCR amplification and sequencing of *ERG11*

Total genomic DNA was extracted from overnight cultures using the DNeasy Plant Mini Kit (Qiagen, Valencia, CA, USA) according to the manufacturer’s recommendations and used as the template for polymerase chain reaction (PCR) amplification of the *ERG11* gene. The primers and conditions used for amplification and sequencing of *ERG11* were described previously as shown in Table 1 [14]. Nucleotide sequences of the amplicon were aligned with the sequence of *C. tropicalis* MYA-3404 (GenBank accession number XM_002550136).

**Table 1:** Primers used in this study

Gene	DNA sequence (5′ to 3′)	Amplicon size (bp)	Reference
*ERG11* for amplification	F:TGAAGAATATCCCACAGGCT	1846	This study
	R:CTTAGCAAGAACTTCTAATGTT		
*ERG11* for RT-PCR	F:GAGATTTGATTGATTCCTTGTTGGT	163	This study
	R:TGTGGTTGTTCAGCCAAATGC		
*CDR1*	F:CCAGAGGTTTGGATTCCGCT	186	This study
	R:TGGCTTTGTCTGCTTTCCCA		
*MDR1*	F:GGGTGCATCATTCCAGCCTA	189	This study
	R:GGGATGGCAATCATCACGAG		
*UPC2*	F:GAGTGGAACAACAACACAACAA	208	This study
	R:TAAATCCCCTAAACCTGAAAGA		
*ACT1*	F:TTTACGCTGGTTTCTCCTTGCC	322	[14]
	R:GCAGCTTCCAAACCTAAATCGG		

### Multilocus sequence typing

Multilocus sequence typing (MLST) was performed as described on the PubMLST website (https://pubmlst.org/ctropicalis/). Each isolate was characterized as a diploid sequence type (DST) based on the sequence of six housekeeping genes [16]. Novel allelic profiles and DSTs were submitted to the *C. tropicalis* MLST database.

### Relative quantification of gene expression

Total RNA extraction and real-time reverse transcription-PCR (RT-PCR) for the evaluation of expression levels of *CDR1*, *MDR1*, *ERG11*, and *UPC2* were performed as descried previously [14, 17]. Total RNA was extracted from strains grown in yeast peptone dextrose media at mid exponential (log) phase using the RNeasy mini kit (Qiagen) according to the manufacturer’s instructions. Total RNA was quantified using a NanoDrop 2000C (Thermo Scientific) and reverse transcription was performed using PrimeScript™ RT Master Mix (Takara, Tokyo, Japan). Quantitative RT-PCR was performed in triplicate using an ABI 7500 Real-Time PCR System (Applied Biosystems, Foster City, CA, USA), SYBR® *Premix Ex Taq*™ II (Takara), and the primers listed in Table 1. The mRNA expression levels of target genes were normalized to the *ACT1* gene using the relative standard curve method as described previously [14]. The transcript levels of fluconazole-resistant isolates were compared with the average expression level of the 10 fluconazole susceptible isolates.

### Statistical analysis

All statistical analyses were performed using SPSS version 24.0 (IBM Corp., Armonk, NY, USA). Continuous variables were compared using the Mann-Whitney U test. *P* values < 0.05 were considered statistically significant.

## Results

### Antifungal susceptibility

Of the 22 *C. tropicalis* isolates, seven were determined to be high-level fluconazole-resistant isolates (MIC, ≥128 mg/L), five were low-level fluconazole-resistant isolates (MIC, 8−64 mg/L), and 10 were control isolates (MIC, ≤2 mg/L). After applying species-specific epidemiological cutoff values, three and twelve isolates were categorized as non-wild-type for itraconazole and posaconazole, respectively. Therefore, a total of three isolates (Ct07R, Ct09R, and Ct10R) had a non-wild-type phenotype or were resistant to the four azole antifungals tested. In addition, all isolates were sensitive to amphotericin B and 5-flucytosine. The results of antifungal susceptibility testing are shown in Table 2.

**Table 2:** Amino acid substitutions, antifungal agent MICs, and patient’s characteristics for 22 *Candida tropicalis* clinical isolates

Designation	Origin	Date of isolation	Age /Sex	Underlying disease	Ward	Substitution of amino acid residue (Erg11p)	DST	Antifungal agent MIC (mg/L)						Previous azole use^a^	Antifungal therapy	Thirty-day outcome
								FLU	VOR	ITR	POS	AMB	5FC			
Ct01R	Blood	8/5/15	37/M	ALL	Hematology	Y132F, S154F	225	128	4	0.25	0.25	0.5	≤0.06	FLU, VOR	CAS, AMB	Survival
Ct02R	Pleural fluid	10/4/14	65/M	Liver Transplantation	ICU	None	639	8	0.5	0.25	0.25	0.5	≤0.06	VOR	None	Death
Ct03R	Blood	9/12/13	72/F	Chronic Renal Failure	Gastroenterological Surgery	Y132F, S154F	639	256	4	0.5	0.5	0.5	0.25	None	FLU, AMB	Death
Ct04R	Blood	11/8/13	26/M	ALL	Hematology	Y132F, S154F	329	64	2	0.25	0.25	0.5	≤0.06	FLU	ITR	Survival
Ct05R	Blood	2/22/15	40/M	CML	Hematology	Y132F, S154F	615	128	4	0.25	0.5	0.5	0.12	FLU, ITR	CAS	Survival
Ct06R	Blood	4/6/17	38/M	AML	Hematology	Y132F, S154F	NT	128	4	0.5	0.5	0.5	0.12	FLU, VOR	CAS, AMB	Survival
Ct07R	Urine	9/15/14	87/F	Cholangitis	ICU	Y132F, S154F	225	256	> 8	1	1	0.5	≤0.06	None	None	Survival
Ct08R	Urine	7/22/15	19/M	Nephrlithotomy	Urology	Y132F, S154F	225	64	4	0.5	1	1	≤0.06	None	VOR	Survival
Ct09R	Urine	8/7/15	64/M	Pneumonia	Respiratory	Y132F, S154F	225	> 256	8	1	1	1	≤0.06	None	None	Survival
Ct10R	Blood	9/2/15	25/F	ALL	Hematology	Y132F, S154F	506	> 256	8	1	1	1	≤0.06	ITR	CAS	Survival
Ct11R	Urine	9/26/15	80/M	Pneumonia	ICU	Y132F, S154F	508	64	2	0.25	0.25	0.5	≤0.06	None	None	Death
Ct12R	Urine	6/17/15	83/F	Adrenocortical Carcinoma	ICU	Y132F, S154F	508	64	4	0.25	0.25	0.5	≤0.06	None	None	Death
Ct01S	Cervix	4/5/17	34/F	Premature Delivery	Obstetrics	None	NT	2	0.12	0.12	0.12	0.25	≤0.06	None	None	Survival
Ct02S	Sputum	6/15/17	62/M	Respiratory Failure	Respiratory	None	NT	1	0.12	0.12	0.12	0.25	≤0.06	None	None	Survival
Ct03S	Urine	6/23/17	16/F	Ewing’s Sarcoma	Musculoskeletal Tumor	None	NT	1	0.06	0.12	0.12	0.25	0.12	None	FLU	Survival
Ct04S	Catheter	6/26/17	44/M	Hepatocellular Carcinoma	ICU	None	NT	2	0.12	0.25	0.25	0.25	≤0.06	None	None	Death
Ct05S	Blood	11/27/14	50/M	Cirrhosis	Hepatology	None	519	2	0.12	0.25	0.25	1	≤0.06	None	CAS	Survival
Ct06S	Blood	2/13/15	43/M	AML	Hematology	None	169	1	0.12	0.25	0.5	0.5	≤0.06	None	ITR	Death
Ct07S	Urine	7/3/15	80/M	CHD	Cardiology	None	343	1	0.12	0.25	0.25	0.5	0.25	None	None	Survival
Ct08S	Blood	9/25/15	42/M	AML	Hematology	None	399	1	0.12	0.25	0.12	1	0.12	None	VOR, AMB	Death
Ct09S	Blood	10/31/14	51/F	AML	Hematology	None	300	2	0.25	0.25	0.25	0.5	0.12	None	VOR, AMB	Death
Ct10S	Urine	7/26/17	23/M	HPS	ICU	None	169	1	0.12	0.25	0.25	0.5	≤0.06	None	None	Survival

*5FC* 5-flucytosine, *ALL* acute lymphocytic leukemia, *AMB* amphotericin B, *AML* acute myeloid leukemia, *CAS* caspofungin, *CHD* coronary heart disease, *CML* chronic myeloid leukemia, *FLU* fluconazole, *HPS* hemophagocytic syndrome, *ITR* itraconazole, *NT* untyped, *POS* posaconazole, *VOR* voriconazole

^a^within 3 months prior to the detection of *C. tropicalis*

### *ERG11* gene mutations

Sequence analysis of the *ERG11* gene revealed no missense mutations in any control isolate. In contrast, two missense mutations, Y132F and S154F, were observed in all fluconazole-resistant isolates except isolate Ct02R (Table 2).

### Multilocus sequence typing analysis

The 10 control isolates and 12 fluconazole-resistant isolates were grouped into nine and seven DSTs, respectively (Table 2). Fluconazole-susceptible isolates were more genetically diverse than fluconazole-resistant isolates. Four fluconazole-resistant isolates belonged to DST225. Two isolates belonged DST169, DST508, and DST639. In addition, the sequences of five isolates were not found in the *C. tropicalis* DST database and the allelic profiles and DSTs of these isolates were submitted to the *C. tropicalis* MLST database.

### Expression levels of *ERG11*, *UPC2*, *CDR1*, and *MDR1*

**Fig. 1:**
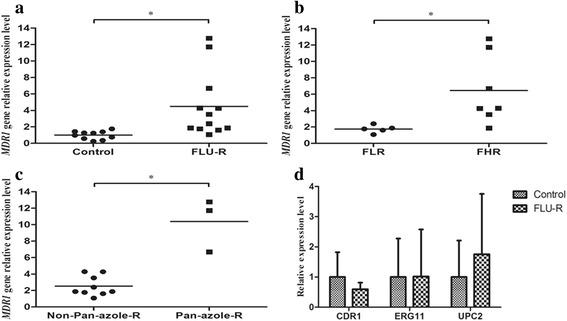
Expression levels of the *MDR1, CDR1*, *ERG11*, and *UPC2*gene in 22 *C. tropicalis* clinical isolates. **a** *MDR1* gene expression levels were higher in fluconazole-resistant (FLU-R) isolates than in control isolates. **b** *MDR1* gene expression levels were higher in high-level fluconazole-resistant (FHR) isolates than in low-level fluconazole-resistant (FLR) isolates. **c** *MDR1* gene expression levels were higher in pan-azole-resistant (Pan-azole-R) isolates than in non-pan-azole-resistant isolates (Non-Pan-azole-R). **d** No significant difference was found in the expression levels of the *CDR1*, *ERG11,* or *UPC2* genes between fluconazole-resistant (FLU-R) and control isolates. Error bars indicate standard deviations. *P < 0.05

Quantitative RT-PCR experiments revealed that expression levels of *MDR1* were higher in the 12 fluconazole-resistant isolates than in the 10 control isolates. Moreover, seven high-level fluconazole resistant isolates had expression levels of *MDR1* than five low-level resistant isolates. In addition, three pan-azole resistant isolates (Ct07R, Ct09R, and Ct10R) had higher expression levels of *MDR1* than the other nine fluconazole-resistant isolates (Fig. 1a, b, c). In contrast, no significant difference was observed in the expression levels of *ERG11*, *UPC2*, or *CDR1* between the 12 fluconazole-resistant isolates and the 10 control isolates (Fig. 1d).

### Clinical information

Clinical information was gathered from chart reviews. Previous use of azole antifungals, history of antifungal therapy, and 30-day outcomes of all *C. tropicalis* infected patients are shown in Table 2. Of the 12 inpatients with fluconazole-resistant *C. tropicalis*, six had been exposed to azole within 3 months prior to the isolates being recovered. Four patients died from any cause in the 30 days after the detection of *C. tropicalis* isolates. In contrast, among the 10 patients from whom fluconazole-susceptible *C. tropicalis* isolates were recovered no patients had previously been exposed to azole and four patients died.

## Discussion

*C. tropicalis* has received widespread attention in recent years due to an increase in fluconazole resistance and the high mortality rate associated with infections [7, 18]. In a global surveillance study conducted in 2013, 31.8% of non-susceptible *C. tropicalis* isolates worldwide were obtained from mainland China [19]. Moreover, the rate of fluconazole resistance of *C. tropicalis* increased from 11.2 to 42.7% between 2011 and 2015 in China [7]. However, understanding of the mechanisms underlying azole resistance in clinical *C. tropicalis* isolates is insufficient compared to that of other *Candida* species.

Studies have demonstrated that Erg11p amino acid substitutions in the 14α-sterol demethylase involved in the biosynthesis of sterol ergosterol results in decreased fluconazole susceptibility in *C. tropicalis* clinical isolates [9, 20, 21]. Jiang et al. demonstrated that two mutations, Y132F and S154F, are responsible for azole resistance by inserting them into wild-type isolate [14]. Similarly, the amino acid substitutions Y132F and S154F in Erg11p were found in all fluconazole-resistant isolates except one (Ct02R: MIC, 8 mg/L) in our study.

A previous study confirmed that the *UPC2* gene, which encodes a global transcriptional regulator of the *ERG11* gene, was up-regulated in azole-resistant *C. tropicalis* isolates [17]. Therefore, we examined *UPC2* gene expression levels in 12 fluconazole-resistant isolates. However, we found that there was no difference in the expression of the *UPC2* gene between fluconazole-resistant and control isolates. These results suggest that *ERG11* overexpression, which results in up-regulation of target enzymes, is not involved in azole resistance in fluconazole-resistant isolates.

In addition to the targeted mutations and overexpression of the enzyme Erg11p, we found that overexpression of the efflux pumps Mdr1p and Cdr1p, encoded by the *MDR1* and *CDR1* genes, are involved in *C. tropicalis* azole resistance [22]. Consistent with this finding, pan-azole resistant *C. tropicalis* isolate, which overexpresses the *MDR1* gene and contains the Y132F and S154F amino acid substitutions in Erg11p, has reportedly been isolated from a patient with acute lymphoblastic leukemia [8]. In the present study, we found that fluconazole-resistant isolates had significantly higher expression levels of the *MDR1* gene than control isolates. Furthermore, high-level fluconazole-resistant isolates had higher levels of *MDR1* expression than low-level fluconazole-resistant isolates. Most importantly, among the 12 fluconazole-resistant isolates, *MDR1* expression levels were highest in the three pan-azole-resistant isolates. Hence, we conclude that overexpression of the *MDR1* gene is associated with high-level fluconazole resistance in *C. tropicalis* clinical isolates. Additionally, these results indicate that the combination of *MDR1* gene overexpression and the amino acid substitutions Y132F and S154F in Erg11p is an important mechanism of pan-azole resistance in *C. tropicalis* clinical isolates.

Of the 12 fluconazole resistant isolates obtained, four belonged to DST225, including two pan-azole resistant isolates (Ct07R and Ct09R). Interestingly, human and fruit samples have recently been found to contain DST225 *C. tropicalis* in Taiwan and these isolates have been shown to be less susceptible to azole drugs [23]. This suggests that *C. tropicalis* fluconazole resistance is related to specific genotypes, such as DST225.

## Conclusion

In conclusion, specific mutations in the *ERG11* gene and upregulation of drug efflux pumps are the most common mechanisms underlying azole resistance in *C. tropicalis*. This study finds that the *MDR1* gene may have a role in determining a high-level of fluconazole resistance and pan-azole resistance in *C. tropicalis* clinical isolates. To the best of our knowledge, this is the first study investigating the relationship between *MDR1* gene overexpression and high-level fluconazole resistance. However, further studies are needed to identify the mechanisms by which *MDR1* is overexpressed. Homologs of gain-of-function mutations in the *C. parapsilosis MRR1* gene, a regulator of the *MDR1* gene [24], also need to be further examined in the future.

## Abbreviations

DST: Diploid sequence type
MALDI-TOF: Matrix-assisted laser desorption/ionization
MIC: Minimum inhibitory concentration
MLST: Multilocus sequence typing
MS: Mass spectrometry
PCR: Polymerase chain reaction
RT-PCR: Real-time reverse transcription-PCR
